# Clinical neuropathology practice guide 06-2012: MGMT testing in elderly glioblastoma patients – yes, but how? 

**DOI:** 10.5414/NP300576

**Published:** 2012-10-16

**Authors:** Anna S. Berghoff, Matthias Preusser

**Affiliations:** 1 Institute of Neurology,; 2 Department of Medicine I, Medical University of Vienna,; 3 Comprehensive Cancer Center Vienna – CNS Tumors Unit, Vienna, Austria

**Keywords:** MGMT, glioblastoma, analytical performance, clinical performance

## Abstract

Abstract. In 2005, a seminal paper showed that glioblastoma patients aged 18 to 70, whose tumors have a methylated MGMT promoter have a better prognosis than patients with tumors carrying an unmethylated MGMT promoter. As a consequence of this and several confirmatory studies, routine MGMT testing in the clinical setting was promoted. However, only few centers have indeed implemented routine clinical MGMT testing, mostly due the lack of clear clinical consequence and because of considerable technical issues with the testing itself. Recently published results of trials on elderly patients with malignant gliomas have revived the call for routine MGMT testing for clinical decision making. These studies strongly support that MGMT status is a predictive factor for response to temozolomide treatment in elderly patients with malignant astrocytic gliomas and its use for therapy decisions could improve patient management, avoid treatment toxicities and save costs. However, although a number of different protocols for MGMT testing from routinely collected and formalin-fixed and paraffin-embedded tissue have been suggested, there is still no commonly accepted test method with sufficient analytical performance. Protocols established in high-throughput specialized academic or commercial laboratories may not be easily transferable to less specialized laboratories. Thus, before MGMT testing can be used and recommended for clinical decision making, an adequate test method with confirmed high repeatability and reproducibility needs to be identified. To this end, specifically designed investigations including stringently controlled interlaboratory ring trials are needed. Such studies need to take into account the considerable variation in pre-analytical tissue handling (e.g., tissue fixation conditions) between laboratories.

## Background 

O6-methylguanine-methyltransferase (MGMT) is a DNA-repair protein that is thought to counteract the effect of alkylating chemotherapy by removing methyl groups from the O6-position of guanine [1]. In line with this assumption an influence of the MGMT promoter methylation status on the outcome of patients with glioblastoma treated with the alkylans temozolomide has repeatedly been observed [2, 3, 4, 5, 6, 7]. In 2005, Hegi et al. [2] demonstrated that glioblastoma patients with intratumoral MGMT gene silencing by promoter hypermethylation had a statistically significantly better outcome when treated with combined radiochemotherapy with temozolomide as compared to patients treated with radiotherapy alone. Patients with unmethylated MGMT promoter, however, had a smaller and statistically non-significant benefit from the addition of temozolomide to radiotherapy. These data were generated from a post-hoc analysis of glioblastoma patients aged 18 – 70 treated in a prospective randomized therapy trial that lead to the definition of adjuvant radiochemotherapy with temozolomide as standard of care [8]. The results of Hegi et al. and a number of subsequent studies confirming the influence of MGMT promoter methylation status on outcome of glioblastoma patients lead to promotion of MGMT testing in the routine clinical setting and also to the launch of several trials with MGMT status as eligibility or stratification criterion [2, 4, 9, 10]. However, although most centers attempted to establish MGMT promoter methylation testing and used it for scientific purposes, only few centers adopted it for routine clinical use [11]. The main reasons were that first, the MGMT status did not allow direct conclusions for patient management, as the available data did not clearly support withholding temozolomide from patients with unmethylated MGMT promoter in the absence of effective alternative treatments; and second, it became soon clear that MGMT testing is technically not trivial and associated with considerable intra- and interlaboratory variability in test results [12]. The most commonly used method (methylation specific polymerase-chain reaction = MSP) was reported to be limited by the adverse influence of formalin-fixation and paraffin-embedding on bisulfite modification, an essential step of the assay [12]. A fairly large number of papers reported on modifications of this technique or alternative methods for MGMT testing to with the goal to overcome this problem, but a consensus on a specific protocol reliably yielding high quality test results was not reached so far [2, 13]. 

## What’s new? 

The results from recently completed and published studies made clear that the MGMT status is of particular interest in elderly patients with high-grade gliomas: 

A study of the German Glioma Network studied a prospectively collected cohort of 233 glioblastoma patients aged 70 or older and found that patients with MGMT methylated tumors had longer progression-free survival when treated with radio- and chemotherapy or chemotherapy only as compared to patients treated with radiotherapy alone. There was no significant benefit of adding chemotherapy to radiotherapy in patients with unmethylated MGMT promoter [4]. 

The Nordic Glioma Study randomized 291 patients with newly diagnosed glioblastoma older than 60 years to receive temozolomide, hypofractionated radiotherapy or standard radiotherapy. Patients treated with temozolomide with MGMT-methylated tumors had significantly longer survival times than patients with unmethylated MGMT promoter and among patients treated with radiotherapy there was no significant difference in outcome according to MGMT status [14]. 

The NOA-08 trial compared a dose-dense temozolomide regimen with radiotherapy alone in 373 elderly patients (age over 65) with anaplastic astrocytoma or glioblastoma. In that trial patients with methylated MGMT promoter had longer event-free survival with temozolomide treatment alone as compared to patients treated with irradiation alone, while patients with unmethylated MGMT promoter fared better with radiotherapy alone [9]. 

Thus, there is compelling evidence that the MGMT status is a predictive factor in elderly patients with malignant astrocytic gliomas and every attempt should be made to implement this information into the day-to-day clinical patient care, as patient allocation to radiotherapy or chemotherapy based on MGMT status could improve patient outcomes, avoid treatment toxicities and save costs. These findings have recently revived the call for routine MGMT testing for clinical decision making. 

## The question remains: how to test? 

A multitude of MGMT assays focusing on the protein, RNA and DNA levels exist. For immunohistochemistry, a poor reliability and high interobserver reliability in interpretation of test results has been demonstrated, thus making this method useless for clinical MGMT testing [12, 15]. Other protein based assays such as Western Blot or MGMT activity assays require unfixed material that is usually not available in the clinical setting [16]. The same holds true for most RNA-based MGMT test methods. Among DNA-based methods, MSP in several variations, pyrosequencing and multiplex-ligation assay (MLPA) among others have been suggested to meet the criteria for clinical use [16]. However, no generally accepted method has emerged so far and there is a lack of studies specifically investigating the advantages and disadvantages of the different MGMT testing protocols and their applicability in different laboratory settings. For example, the adoption of protocols successfully used in experienced high-end laboratories with high sample through-put to pathology laboratories with fewer samples and less specialized infrastructure and personnel may be problematic. Furthermore, there is considerable interlaboratory variation in pre-analytical tumor tissue handling, e.g., tissue fixation conditions, and definition of standards could facilitate not only MGMT testing for clinical purposes but also other molecular investigations [17]. 

In sum, we would like to emphasize that for widespread use of MGMT testing for clinical decision making, a robust and reliable method is needed and there is an urgent need to identify a technique that fulfills all criteria of high repeatability and reproducibility and can be implemented easily in standard laboratories [18]. 

## Acknowledgment 

The authors thank Professor Martin van den Bent (Rotterdam) for critical review of this manuscript. 
